# Post-Transplant Pain and Paralysis: Neurologic Amyotrophy as an Atypical Cause of Shoulder Dysfunction Following Hematopoietic Stem Cell Transplant

**DOI:** 10.3390/cancers17111816

**Published:** 2025-05-29

**Authors:** Franchesca König, Chanel Davidoff, Katarzyna Ibanez, Sinchun Hwang, Ilan Goldstein, Darren R. Feldman, Boglarka Gyurkocza, Sergio A. Giralt, Ioannis Politikos, Doris M. Ponce, Michael Scordo, Grigory Syrkin, Christian M. Custodio, Gunjan L. Shah

**Affiliations:** 1 Department of Physical Medicine & Rehabilitation, University of Colorado Anschutz Medical Campus, Aurora, CO 80045, USA; franchesca.konig@cuanschutz.edu; 2 Department of Physical Medicine and Rehabilitation, Lenox Hill Hospital—Northwell Health, New York, NY 10075, USA; cdavidoff@northwell.edu; 3 Rehabilitation Medicine Service, Department of Neurology, Memorial Sloan Kettering Cancer Center, New York, NY 10065, USA; ibanezk@mskcc.org (K.I.); gsyrkin@montefiore.org (G.S.); custodc1@mskcc.org (C.M.C.); 4 Department of Radiology, Memorial Sloan Kettering Cancer Center, New York, NY 10065, USA; hwangs@mskcc.org; 5 Adult Bone Marrow Transplant Service, Department of Medicine, Memorial Sloan Kettering Cancer Center, New York, NY 10065, USA; goldsti3@mskcc.org (I.G.); gyurkocb@mskcc.org (B.G.); giralts@mskcc.org (S.A.G.); politikosi@mskcc.org (I.P.); ponced@mskcc.org (D.M.P.);; 6 Genitourinary Service, Department of Medicine, Memorial Sloan Kettering Cancer Center, New York, NY 10065, USA; feldmand@mskcc.org

**Keywords:** neuralgic amyotrophy, parsonage turner syndrome, brachial plexus, shoulder pain, hematopoietic stem cell transplant

## Abstract

Background: Neuralgic amyotrophy (NA), also known as Parsonage–Turner syndrome, brachial neuritis, and idiopathic brachial plexopathy, is a rare and potentially debilitating peripheral nerve disorder characterized by acute-onset shoulder pain followed by progressive motor deficits. It is often under-recognized, with an estimated incidence of 1 to 3 per 100,000 annually, though some studies suggest the actual prevalence may be significantly higher. The condition typically progresses through three phases, an acute painful phase, a phase of weakness, and a recovery phase, with sensory disturbances common in addition to motor weakness. The exact pathogenesis of NA remains unclear, though it is thought to involve a combination of genetic, environmental, and immunological factors. While neurologic complications following hematopoietic stem cell transplantation (HSCT), such as neuropathies and myopathies, have been documented, NA remains exceedingly rare in this context, with only a few reported cases. The pathophysiology in HSCT patients is hypothesized to involve immune dysregulation, graft-versus-host disease (GvHD), infection, and the effects of immunosuppressive therapy. Diagnosis is primarily clinical, supported by electrodiagnostic studies and MRI, though no laboratory markers exist. The management of NA is largely supportive and multimodal, focusing on pain control and rehabilitation. Objectives: The objective of this study was to describe the characteristics, clinical course, and outcomes of patients admitted for HSCT who were subsequently diagnosed with NA. Study Design: This retrospective case series from a single institution examined nine (N = 9) patients who developed acute shoulder pain following HSCT. We collected data on demographics, transplant details, clinical features, MRI findings, and electrodiagnostic studies, summarized using descriptive statistics. The diagnosis of neurologic amyotrophy was based on clinical presentation and corroborated by imaging and electrodiagnostic results. Long-term follow-up was assessed to evaluate symptom recovery. Results: Between August 2020 and July 2022, nine patients (44% male, median age 60) were diagnosed with NA following autologous (n = 4) or allogeneic (n = 5) HSCT. The onset of severe shoulder pain occurred at a median of 9 days post-transplant (range 1–21 days), with the majority of patients experiencing unilateral pain, predominantly affecting the right shoulder (55%). Neurologic weakness developed on average 5.1 days after pain onset, and sensory deficits were observed in all but one patient. MRI findings revealed muscle edema, atrophy, and enhancement in six patients, while electromyography confirmed NA in five. Due to the small sample size, statistical analyses, including *p*-values, confidence intervals, and trend comparisons, were not performed, and thus no conclusions can be drawn regarding associations between variables such as early onset and worse outcomes. Shoulder pain resolved after a median of 23 days (range 8–40 days). Long-term follow-up (>1 year) showed that three patients achieved full or near-full recovery, four partially recovered, and two showed minimal improvement. **Conclusions**: NA should be highly suspected in patients with acute shoulder pain and neurologic symptoms post-HSCT. To improve diagnostic accuracy and clinical outcomes, we recommend enhanced clinician awareness, the implementation of targeted diagnostic protocols (such as MRI and electrodiagnostic studies), and the establishment of standardized long-term follow-up protocols.

## Simple Summary

This study describes a rare nerve condition called neuralgic amyotrophy, which causes sudden shoulder pain followed by weakness, in patients with blood cancers who have undergone a type of treatment called stem cell transplant. Although this condition is uncommon, it can be serious and is often missed by doctors because its symptoms resemble other problems. We looked back at the records of nine patients treated at our hospital who developed this condition shortly after receiving a stem cell transplant. Most of them experienced sharp shoulder pain within a few days of the transplant, followed by weakness and numbness the arm. Tests like magnetic resonance imaging (MRI) and nerve studies helped confirm the diagnosis. While some patients fully recovered, others continued to have long-term weakness or pain. Because this condition is difficult to diagnose and manage, our findings highlight the need for greater awareness among healthcare providers. By recognizing the symptoms early and using the right tests, docto.

## 1. Introduction

Neuralgic amyotrophy (NA), known by a variety of terms including Parsonage–Turner syndrome (PTS), brachial neuritis, and idiopathic brachial plexopathy, is a rare and potentially debilitating peripheral nerve disorder characterized by acute-onset pain and progressive motor deficits [1,2]. Although first formally described by Drs. Parsonage and Turner in the Lancet in 1948 [3], earlier research mentions similar cases as far back as 1897 [1]. Its true prevalence is difficult to estimate with precision, largely due to under-recognition. Historically, the estimated incidence of NA has been reported as between 1 and 3 per 100,000 annually [2,4,5]. However, one study suggests that the actual prevalence may be significantly higher—potentially 30 to 50 times greater—implying a substantial burden of unrecognized or misdiagnosed disease [2].

NA may manifest with a broad range of phenotypes; however, the classic presentation begins with an abrupt onset of intense, unilateral shoulder pain followed by the development of progressive, painless muscle weakness [1,2]. NA typically progresses through three phases: the painful phase, the phase of weakness, and the recovery phase. In about 90% of cases, pain is the initial symptom, characterized as acute, atraumatic, and severe, often rated ≥ 7/10 on the visual analog scale [6]. The duration of the painful phase is variable, ranging from a single day to 2 months, though the average is 7–14 days [7]. Motor weakness usually emerges within a few days to weeks after the onset of pain, although it may be overlooked initially due to the severity of the pain, which discourages movement of the affected limb. Sensory disturbances, such as numbness or paresthesia, also occur. Interestingly, the topography of pain does not always correspond with areas of motor weakness or sensory disturbance, adding to the complexity of diagnosis [2]. This discordance highlights the importance of maintaining a high index of suspicion, particularly when patients present with unexplained pain and evolving neurologic symptoms. The third phase of NA is the recovery phase, which may span months to years depending on the extent of nerve damage and subsequent innervation. Some studies indicate that 80–90% of patients experience favorable recovery within two to three years [8]. Other studies, however, highlight persistent deficits and suboptimal function, with continued long-term complaints of pain and fatigue, with half to two-thirds experiencing impairments in their activities of daily living [6,9,10].

The precise pathogenesis of NA remains elusive, though it is thought to be multifactorial. The most widely accepted hypothesis involves a complex interplay of environmental, mechanical, and individual factors [2]. Several conditions are associated with its development, including viral and bacterial infections, surgery, strenuous exercise, immunizations, trauma, and pregnancy or childbirth [1,11]. A hereditary form also exists, which appears to predispose individuals to recurrent episodes [7].

Neurologic complications affecting the peripheral nervous system, including neuropathies, myopathies, and neuromuscular junction disorders, have been documented following hematopoietic stem cell transplantation [12,13,14,15,16]. While NA has been documented across a broad spectrum of clinical contexts, its occurrence following hematopoietic stem cell transplantation is exceedingly rare, with only a handful of published case reports to date [17,18,19,20]. The exact pathophysiology in patients undergoing HSCT remains unclear, but it is hypothesized that several interrelated factors may contribute. For one, immune dysregulation, involving the activation of immune cells against host tissues, can trigger autoimmune responses, contributing to neurologic manifestations [21]. Additionally, patients undergoing HSCT are highly susceptible to infections due to prolonged immunosuppression, which can trigger an inflammatory response and lead to neurologic dysfunction [22]. There may also be neurological manifestations of graft-versus-host disease, where donor immune cells attack the recipient’s tissues. Lastly, immunosuppressive agents can alter immune responses and potentially trigger autoimmune neuropathies [23]. Notably, most reported cases occur after autologous rather than allogeneic transplants [17], though the precise incidence of NA in this population remains unknown.

The diagnosis of NA remains clinical at its core and is based on a thorough history and neuromuscular physical examination. Electrodiagnostic studies (EDX) may serve as a complementary tool in confirming the diagnosis, although it should be noted that testing is optimally performed no earlier than four weeks after the onset of the injury in question to allow for the detection of denervation changes [24]. Typical EDX findings include denervation and/or reinnervation, prolonged motor latency, and decreased sensory nerve action potential and compound motor action potential amplitudes in the setting of normal sensory conduction velocity [25,26]. Magnetic resonance imaging (MRI) may also offer diagnostic value. The MRI of the brachial plexus may be obtained in addition to an MRI of the cervical spine if a nerve root disorder is being considered or an MRI of the shoulder if intra-articular pathology is on the differential. Magnetic resonance neurography (MRN) and more recently ultrasonography may provide additional insight with the presence of hourglass-like constrictions of nerves and/or nerve fascicles [27,28,29]. Notably, there are no known laboratory findings to support the diagnosis of NA.

There is currently no established standard of care for the management of NA. A systematic Cochrane review of the existing literature on conservative management for NA concluded that there is no evidence from randomized controlled trials supporting the efficacy of any specific treatment modality [30]. In clinical practice, management is typically supportive and multimodal. Common interventions include analgesic therapies for pain control, particularly in the initial painful phase. Anecdotally, high-dose steroids have been proposed as initial management with some evidence to support decreased duration of acute pain [31]. Other medications such as acetaminophen and gabapentinoids may also be explored. Pain management is crucial to allow for timely rehabilitation. Similarly, there is no consensus on specific therapeutic interventions for NA; however, it is generally accepted that rehabilitation should prioritize shoulder-focused therapy, including range of motion exercises to prevent joint stiffness and contracture, scapular stabilization, functional motor control, and progressive strengthening [31]. Precautions, especially specific to this patient population such as thrombocytopenia, should be taken into account.

In light of the limited literature on NA following HSCT, the aim of this study was to describe the clinical characteristics, temporal course, and outcomes of patients who underwent HSCT and were subsequently diagnosed with neuralgic amyotrophy. In particular, the study sought to explore the demographic and clinical features of this population, examine the timing and progression of neurological symptoms following HSCT, and assess both short- and long-term outcomes associated with NA in this context.

## 2. Materials and Methods

### 2.1. Study Design and Sample Population

This single-center retrospective case series was conducted to describe the characteristics, the clinical course, and the outcomes of patients admitted to Memorial Sloan Kettering Cancer Center for allogeneic or autologous HSCT who were subsequently diagnosed with neurologic amyotrophy (NA). The study was approved by the Institutional Review Board (IRB) at our institution and conducted in accordance with ethical guidelines and regulations governing retrospective chart reviews. The requirement for informed consent was waived due to the retrospective nature of the study and the use of de-identified data.

Patients were eligible for inclusion in this retrospective case series if they met both of the following criteria:

1. Underwent autologous or allogeneic hematopoietic cell transplantation at the study institution between August 2020 and July 2022;
2. Developed acute-onset shoulder pain followed by weakness consistent with a clinical diagnosis of neuralgic amyotrophy, as determined by physical examination.

Patients were excluded if they met either of the following criteria:

1. Had alternative neurologic diagnoses explaining their symptoms (e.g., cervical radiculopathy, central nervous system pathology, or mechanical injury);
2. Lacked sufficient clinical documentation to support a diagnosis of NA based on history, examination, and follow-up data.

### 2.2. Data Collection

The medical records of nine adult patients (N = 9) who were admitted for HSCT between August 2020 and July 2022 and subsequently presented with acute-onset unilateral or bilateral shoulder pain accompanied by neurologic symptoms were identified and retrospectively reviewed. Patients were initially identified by the inpatient transplant team, after which an inpatient consultation was requested with the Physical Medicine and Rehabilitation (PM&R) Service for further evaluation and management. The diagnosis of post-HSCT neurologic amyotrophy was established primarily through clinical presentation, with further confirmation obtained from a detailed physical examination and corroborative findings from radiographic imaging and/or electrodiagnostic studies. This comprehensive, multidisciplinary approach ensured a robust and well-supported diagnosis, integrating clinical findings with objective diagnostic evidence.

Key data elements were systematically extracted and analyzed, including patient demographics (age, gender, and oncologic diagnosis) and HSCT details (type and date of HSCT, intensity and types of conditioning regimens, GvHD prophylaxis (where applicable), and engraftment status).

Additionally, clinical characteristics of symptoms of NA were thoroughly documented, including onset, duration and location of pain onset, distribution of neurological deficits, and long-term outcomes. Relevant imaging studies, particularly MRI of the brachial plexus and/or shoulder, as well as available EDX studies, were reviewed. These studies were evaluated by a board-certified radiologist and board-certified electromyographer to ensure accurate interpretation and confirmation of clinical findings.

Due to the retrospective nature of this study, some clinical variables were not available for all patients. Missing data were not imputed. Analyses were conducted using available case data. When relevant, we noted the absence of data in the results and interpreted the findings accordingly.

### 2.3. Statistical Analysis

Descriptive statistics, including medians and frequencies, were used to summarize the collected data. Due to the limited sample size of this retrospective review, statistical analyses, including confidence intervals, *p*-values, and trend comparisons, were not performed, and thus no formal inferential statistics could be applied. As a result, no conclusions regarding potential associations, such as the correlation between early onset and worse outcomes, can be drawn from these data.

## 3. Results

### 3.1. Demographics

Between August 2020 and July 2022, a total of nine patients (44% male, median age 60 years) were diagnosed with NA following HSCT. This study cohort included both autologous and allogeneic HSCT. Specifically, four patients underwent autologous HSCT: two for multiple myeloma, one for non-Hodgkin lymphoma, and one for a germ cell tumor. The remaining five patients received allogeneic HSCT, with indications including myelodysplastic syndrome (n = 3) and acute myelogenous leukemia (n = 2).

The demographic and oncologic characteristics of our study cohort, as well as details of the HSCT procedure, are summarized in Table 1. Table 2 further expands on the treatment regimens received by each individual patient, including specific details of conditioning protocols and engraftment status.

### 3.2. Diagnosis and Clinical Presentation

In our cohort, the majority (67%) of the patients developed symptoms of NA within 10 days following HSCT, with a median onset of 9 days and a range spanning from 1 to 21 days. In terms of symptom localization, the majority of patients experienced unilateral symptoms, with the right shoulder being more frequently affected (right shoulder—55%, left shoulder—22%) and a smaller subset (33%) of patients presenting with bilateral shoulder involvement. Neurologic weakness in the shoulder(s) emerged on average 5.1 days after the onset of pain, with a range of 1 to 15 days. It is worth noting that the majority of the patients (n = 6) experienced neurologic weakness within 3 days of pain onset. Furthermore, sensory deficits were present in all but one patient. Shoulder pain resolved after a median of 23 days, with a range of 8 to 40 days.

Table 3a details the individual clinical characteristics of our patients, while Table 3b provides a summary of the cohort.

### 3.3. Diagnostic Findings

MRI of the shoulder and/or brachial plexus was conducted for all patients as part of the diagnostic workup. A total of four patients underwent imaging of both the shoulder and brachial plexus, while three patients had brachial plexus imaging only. Two patients had an MRI of the symptomatic shoulder only. The purpose of this imaging was to confirm the clinical diagnosis of NA and to rule out other potential diagnoses, such as rotator cuff pathology.

**Figure 1:**
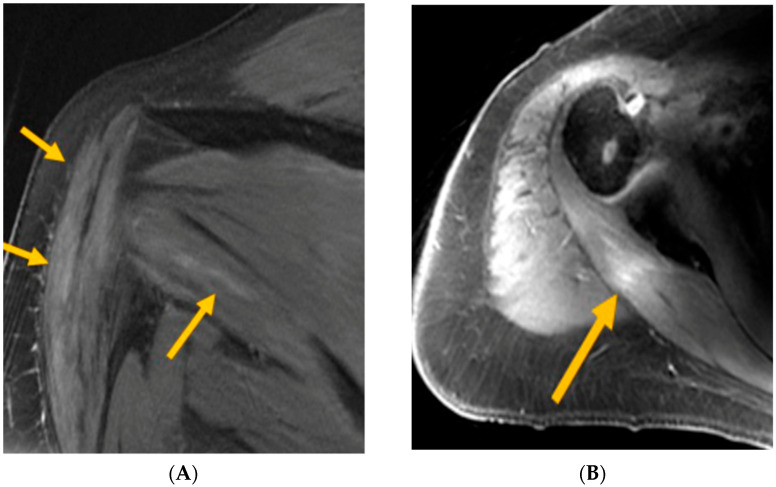
(**A**) MRI T2FS—arrows indicate deltoid and teres minor intramuscular edema (consistent with axillary neuropathy); (**B**) MRI T1FS—arrow indicates post-infraspinatus enhancement (consistent with upper trunk brachial plexopathy).

The MRI findings revealed periscapular muscle edema, atrophy, and/or enhancement in five of the patients, which are suggestive of neurogenic involvement and support the diagnosis of NA. One additional patient exhibited asymmetric prominence and enhancement of the left brachial plexus, though no muscle changes were observed in this case. It is worth noting that in this patient, only brachial plexus imaging was performed, which may account for the lack of visualizable muscle abnormalities. Muscle signal abnormalities, which are suggestive of neurogenic processes, were observed across multiple muscles, including the supraspinatus, infraspinatus, deltoid, teres minor, serratus anterior, and intercostals (Figure 1A,B).

In addition to MRI, electrodiagnostic studies (EDX) were performed on six patients, five of whom demonstrated findings consistent with NA. Two of these patients exhibited axillary neuropathies, one had upper trunk plexopathy, one showed bilateral posterior cord plexopathy, and one presented with diffuse bilateral brachial plexopathy.

### 3.4. Treatment and Long-Term Outcomes

There was no established, standardized treatment protocol for NA within the patient population of this study. The management of NA was therefore individualized, with the primary focus on ensuring adequate analgesic control and facilitating optimal rehabilitative care. Pain management strategies aimed to alleviate the acute, severe discomfort associated with the initial painful phase of NA. Rehabilitative care was a crucial component of the management strategy. PM&R physicians were consulted and played an integral role in not only diagnosing NA but also developing rehabilitation plans tailored to each patient’s specific functional deficits. The goal of rehabilitation was to minimize pain, preserve muscle function, prevent contractures, and improve overall mobility and strength. Skilled therapists, including physical and occupational therapy, were involved in all patients to ensure follow-through with the rehabilitation plan of care.

Outcomes were notably variable, reflecting the unpredictable nature of the disease. One-third of patients achieved complete or nearly complete neurologic recovery within 2 to 12 months. For the remaining patients, recovery was incomplete and more gradual. Four patients experienced partial neurological recovery, with improvements noted within a range of 7 to 24 months. One patient, unfortunately, passed away at 3 months post-onset of NA without demonstrating any neurological recovery. The last patient in the cohort did not exhibit significant neurological improvement at 10 months post-NA onset, at which point he transferred care to a local facility. This patient ultimately succumbed to his oncologic disease 10 months later.

## 4. Discussion

This case series contributes valuable insights to the limited body of literature describing NA in an immediate post-HSCT setting. Despite being a unique and underexplored patient population, our findings underscore the importance of actively investigating the hypothesis that NA in this setting may be, at least in part, driven by immune-mediated and inflammatory mechanisms. This aligns with broader pathophysiologic theories regarding idiopathic NA and raises the possibility that transplant-related immune dysregulation, including subclinical manifestations of graft-versus-host disease, infection-triggered immune responses, or the effects of immunosuppressive therapies, may serve as inciting or amplifying factors in susceptible individuals.

Although the true incidence of NA following HSCT remains unknown, our identification of 9 cases among 841 stem cell transplants (including both allogeneic and autologous) performed over a two-year period suggests a rate higher than that observed in the general population, where annual incidence is estimated at 1–3 cases per 100,000 individuals. This discrepancy highlights a potentially underappreciated burden of disease in post-HSCT patients. Nevertheless, we believe our findings still likely underrepresent the true prevalence of NA in this population. Several factors that may contribute to this underestimation include the medical team’s focus on pain management without considering the diagnosis of NA, the early discharge of patients during the post-HSCT phase, and the underreporting of pain or neurologic symptoms by patients.

Raising awareness within the transplant community will facilitate earlier diagnosis, prompt and appropriate intervention, and improved understanding of NA. The clinical presentation of NA in our cohort followed a classic pattern, characterized by acute, severe pain followed by neurologic weakness as early as within 24 h of onset of pain. This hallmark presentation strongly supports the diagnosis of NA, particularly when corroborated by imaging and EDX studies. Notably, three patients in our cohort had nondiagnostic imaging results; however, EDX confirmed NA in two of these cases, underscoring the diagnostic value of these types of studies. In one case, the diagnosis was made clinically due to the absence of EDX testing.

Despite the insights provided by our study, there are important limitations to consider. The small sample size and retrospective nature of the analysis limit the generalizability of our findings. Diagnostic evaluations were not standardized across patients and were often performed based on clinician judgment rather than a predefined protocol for NA detection. Furthermore, MRI was not uniformly protocoled to detect hourglass-like constrictions, a recognized MRN feature of NA. Similarly, EDX testing was limited due to clinical contraindications such as thrombocytopenia and, ultimately, patient willingness to undergo this uncomfortable testing in the inpatient setting while already in pain.

Efforts to diagnose NA at earlier stages aim to enable timely intervention and potentially alter the disease’s natural progression. However, these proposed interventions include high-dose steroids and intravenous immunoglobulin (IVIG) treatments, which might not be appropriate for our patient population due to associated risks [32]. Further studies are needed to evaluate the safety and efficacy of these treatments in the post-HSCT context.

Given that a considerable proportion of patients with NA experience prolonged or incomplete recovery, long-term follow-up is essential. In our cohort, neurological recovery ranged from full resolution to persistent deficits, with variable trajectories over months. This heterogeneity underscores the need for continued monitoring and personalized rehabilitation strategies. When medically appropriate, neurosurgical consultations should be considered in cases where fascicular constrictions are visualized. Surgical neurolysis, including targeted fascicular release, may lead to subsequent recovery of the involved nerve(s) [11]. Anecdotal evidence suggests that neurolysis may confer functional recovery months or even years after symptoms onset, provided serial EDX testing demonstrates ongoing muscle excitability. For patients with persistent motor deficits and limited recovery despite conservative management, advanced surgical interventions such as nerve transfer procedures may offer additional benefits.

## 5. Conclusions

To summarize, our work aims to fill a critical gap in the current understanding of NA in the setting of HSCT, a patient population in which this condition is likely under-recognized and underreported. By characterizing the clinical features, diagnostic findings, and recovery trajectories, our work lays an initial foundation for enhancing both diagnostic vigilance and clinical management in this patient population. Given the possibly overlapping symptomatology of NA with other pain clinical pictures, maintaining a high index of suspicion for NA is essential. Acute, severe shoulder pain with subsequent motor weakness should prompt timely neurologic evaluation and consideration of NA in the differential diagnosis. Delays in recognition may lead to prolonged functional impairment, missed opportunities for early intervention, and possibly increased patient morbidity.

In light of the hypothesized immune-mediated and inflammatory mechanisms potentially contributing to NA in the HSCT setting, early identification is not only important for symptom management but may also influence future therapeutic strategies. While there is currently no standardized treatment protocol, prompt diagnosis allows for the initiation of pain management, patient education, and rehabilitation.

To improve diagnostic accuracy and clinical outcomes, we propose several key recommendations. First, increased awareness and education among clinicians are needed to better recognize the hallmark features of NA. Second, the use of targeted diagnostic protocols, including MRI and EDX, should be encouraged when appropriate and clinical suspicion arises. Finally, comprehensive long-term follow-up is essential to monitor neurological recovery, adapt rehabilitation strategies, and identify candidates who may benefit from advanced interventions such as surgical neurolysis or nerve transfer procedures. Standardizing follow-up protocols for patients diagnosed with NA post-HSCT will help build a clearer understanding of recovery patterns and inform future prospective studies.

## Figures and Tables

**Table 1:** Patient demographics.

N	9	
Age—median (range)	60 (30–73)	
Sex (male) N (%)	44.4	
Race N (%)	White, non-Hispanic	9 (100)
Malignancy N (%)	AML	2 (22)
	GCT	1 (11)
	MDS	3 (33)
	MM	2 (22)
	NHL	1 (11)
Stem Cell Source (%)	Allo PBSC	5 (55)
	Auto PBSC	4 (44)
Conditioning Regimen N (%)		
	ALLO:	
	Busulfan Fludarabine Melphalan	1 (11)
	Carboplatin Etoposide	1 (11)
	Cyclophosphamide Fludarabine Thio-TEPA TBI	1 (11)
	Fludarabine Melphalan	3 (33)
	AUTO:	
	Carmustine Cytarabine Etoposide Melphalan	1 (11)
	Melphalan	2 (22)

**Table 2:** Treatment profile of individual transplant recipients.

Case	Disease	Stem Cell Source	Status	Conditioning Regimen	Intensity	GvHD Prophylaxis	Engraftment Status
1	MM	Auto PBSC	1st partial response	Melphalan	Ablative	-	Engrafted
2	MM	Auto PBSC	Stable	Melphalan	Ablative	-	Engrafted
3	NHL	Auto PBSC	1st remission	Carmustine/ Cytarabine/ Etoposide/ Melphalan	Ablative	-	Engrafted
4	MDS	Allo PBSC	RAEB-1	Busulfan/ Fludarabine/ Melphalan	Ablative	-	Engrafted
5	AML	Allo PBSC	MRD neg 1st CR	Cyclophosphamide/Fludarabine/ Thio-TEPA/TBI	Reduced Intensity	Methotrexate x4/Tacrolimus	Engrafted
6	GCT	Auto PBSC	Relapse	Carboplatin/ Etoposide	Ablative	-	Engrafted
7	MDS	Allo PBSC	RAEB-2	Fludarabine/ Melphalan	Reduced Intensity	Methotrexate x4/Tacrolimus	Engrafted
8	AML	Allo PBSC	MRD neg 1st CR	Fludarabine/ Melphalan	Reduced Intensity	Methotrexate x4/Tacrolimus	Engrafted
9	MDS	Allo PBSC	RAEB-1	Fludarabine/ Melphalan	Reduced Intensity	Cyclophosphamide/ Mycophenolate Mofetil/Tacrolimus	Engrafted

MM (multiple myeloma); NHL (non-Hodgkin lymphoma); MDS (myelodysplastic syndrome); AML (acute myeloid leukemia); GCT (giant cell tumor); PBSC (peripheral blood stem cell) TBI (total body irradiation); MRD (minimal residual disease); RAEB (refractory anemia with excessive blasts).

**Table 3:** (**a**): Clinical characteristics of individual transplant recipients; (**b**): summary of clinical characteristics.

(a)									
Case	Pain			Weakness		Sensory Symptoms	MRI Findings	EMG Findings	Outcome
	Onset from HSCT (Days)	Distribution	Duration (Days)	Onset from Pain (Days)	Distribution				
1	21	Right shoulder	23	7	Unilateral Shoulder	+	E, A, EC in teres minor	Axillary Neuropathy	Partial Recovery at 24 months
2	9	Right shoulder, forearm	9	3	Unilateral shoulder, elbow	+	Unremarkable	Axillary neuropathy	Complete Recovery at 7 months
3	10	Right shoulder	27	15	Unilateral shoulder	+	E, EC in supra and infraspinatus	Upper trunk brachial plexopathy	Complete Recovery at 13 months, patient deceased *
4	20	Left shoulder, forearm	29	8	Unilateral wrist/hand	+	Possible plexopathy w/o E	Normal	Partial recovery at 26 months
5	1	Bilateral Shoulder	32	1	Bilateral, Diffuse	+	E, EC in Supraspinatus, Infraspinatus, Teres Minor, Deltoid	Bilateral diffuse brachial plexopathy	Partial Recovery at 12 months
6	19	Bilateral Shoulder	40	1	Bilateral shoulder	+	N/A	Bilateral brachial plexopathy, posterior cord	Minimal Recovery at 10 months, patient deceased *
7	5	Left Shoulder, forearm	21	2	Unilateral Shoulder	-	Unremarkable	N/A	Partial Recovery at 7 months
8	2	Bilateral Shoulder	8	3	Bilateral shoulder	+	E, A, EC in Supraspinatus and Infraspinatus	N/A	Minimal Recovery at 3 months, Patient deceased **
9	6	Right shoulder	11	6	Unilateral shoulder, forearm	-	E, EC—serratus ant, intercostals	N/A	Complete recovery at 2 months
**(b)**									
**Pain Onset (Median, Days)**							**9 (1–21)**		
Pain distribution Unilateral, N (%)							6 (66.6)		
Weakness onset avg (Days)							5 (1–15)		
Pain resolution (median, Days)							23 (8–40)		
Sensory symptoms N (%)							7 (77.7)		
Positive MRI findings N (%)							* 6 (75)		
Positive EMG findings N (%)							** 5 (86)		
Recovery Complete at <2 years, N (%)							3 (33.3)		

(**a**): E = edema, A = atrophy, EC = contrast enhancement, N/A = not available (not completed). * Cause of death due to progression of oncologic disease. ** Cause of death due to GvHD. (**b**): * Total 8 MRIs performed. ** Total 6 EMGs performed.

## Data Availability

The data that support the findings of this retrospective case series are not publicly available due to institutional privacy policies and patient confidentiality concerns. De-identified data may be made available from the corresponding author upon reasonable request and with appropriate institutional review board (IRB) approval.
